# Metastatic Primary Gastric Squamous Cell Carcinoma: An Uncommon Presentation of a Rare Malignancy

**DOI:** 10.1155/2019/5305023

**Published:** 2019-10-07

**Authors:** Michael Beattie, Ramy Mansour, Derek Thigpin, Carolyn Haus

**Affiliations:** ^1^Department of Internal Medicine, Genesys Regional Medical Center, 1 Genesys Pkwy, Grand Blanc, MI 48439, USA; ^2^Department of Gastroenterology, Genesys Regional Medical Center, 1 Genesys Pkwy, Grand Blanc, MI 48439, USA; ^3^Department of Pathology, Genesys Regional Medical Center, 1 Genesys Pkwy, Grand Blanc, MI 48439, USA

## Abstract

Primary gastric squamous cell carcinoma is a very rare disease. A 53-year-old male with history of hypertension, alcoholism, and nicotine abuse presented to the hospital after a syncopal episode. He complained of bloating abdominal pain, early satiety, and poor appetite. A CT of his abdomen and pelvis revealed a gastric mass with diffuse hepatic metastasis. A gastric mass was seen on upper endoscopy and biopsies revealed gastric squamous cell carcinoma. There was no involvement of the esophagus. This case should add to the limited literature and serve as a reminder that while this is a rare malignancy, it must be considered when evaluating a gastric mass.

## 1. Introduction

Primary gastric squamous cell carcinoma (PGSCC) accounts for less than 0.2% of all primary gastric carcinomas [1]. The presentation is nonspecific and given its rarity the etiology is not well known. Several strict diagnostic criteria have been established to make the diagnosis [2–5].

## 2. Case Report

A 53-year-old white male with hypertension, chronic alcoholism, and tobacco abuse presented to the emergency department for syncope while standing outside. Review of systems was positive for four weeks of abdominal discomfort and poor oral intake due to increased abdominal pressure. He denied any changes in weight, melena, hematochezia, hematemesis, or dysphagia.

Physical examination revealed a soft, moderately distended abdomen with mild epigastric and right upper quadrant tenderness with guarding. Bowel sounds were hypoactive. There was no supraclavicular lymphadenopathy, and the rest of the exam was unremarkable.

**Figure 1:**
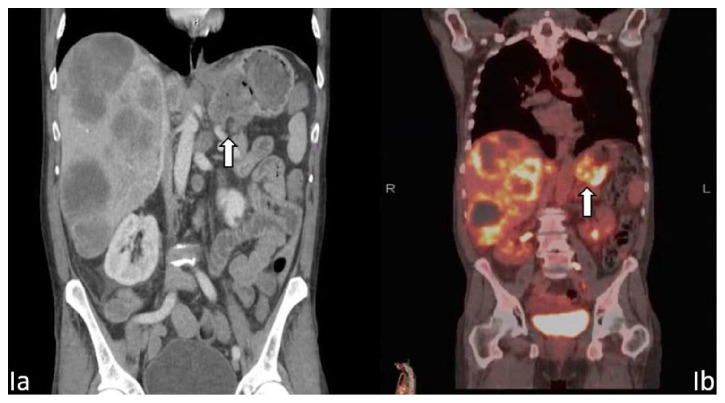
(a) CT demonstrating gastric mass in the lesser curvature (arrow) with diffuse hepatic metastases. (b) PET scan with 6.5 cm hypermetabolic gastric (Arrow) and no additional sources for a primary tumor. Diffuse hepatic metastases again noted.

Initial work-up found the patient to be hyponatremic at 118 mmol/L (136–144 mmol/L), Aspartate Aminotransferase (AST) of 184 U/L (15–41 U/L), Alkaline Phosphatase 390 U/L (40–129 U/L). Gamma-Glutamyltransferase (GGT) was 1080 U/L (7–50 U/L), total bilirubin 1.7 mg/dL (0.3–1.2 mg/dL) with direct bilirubin of 0.9 mg/dL (0.1–0.5 mg/dL). Alanine Aminotransferase (ALT) was within normal limits at 53 U/L (17–63 U/L). Complete blood count (CBC) revealed a leukocytosis of 15.3 K/cmm (4.0–11.0 K/cmm), Hemoglobin 10.9 g/dL (12–17.3 g/dL), Hematocrit 32.3% (36–53%), and a platelet count of 384 K/cmm (140–440 K/cmm). CT of the abdomen and pelvis with IV contrast revealed a 6.1 cm mass lesion in the lesser curvature of the stomach contiguous with the gastrohepatic ligament, as well as extensive hepatic metastatic lesions, with the largest measuring 9.5 cm (Figure 1(a)). Serum CA 19-9 was 54.2 U/mL (0–35.5 U/ml) and Carcinoembryonic Antigen (CEA) 1.6 ng/mL (0–3.0 ng/mL).

**Figure 2:**
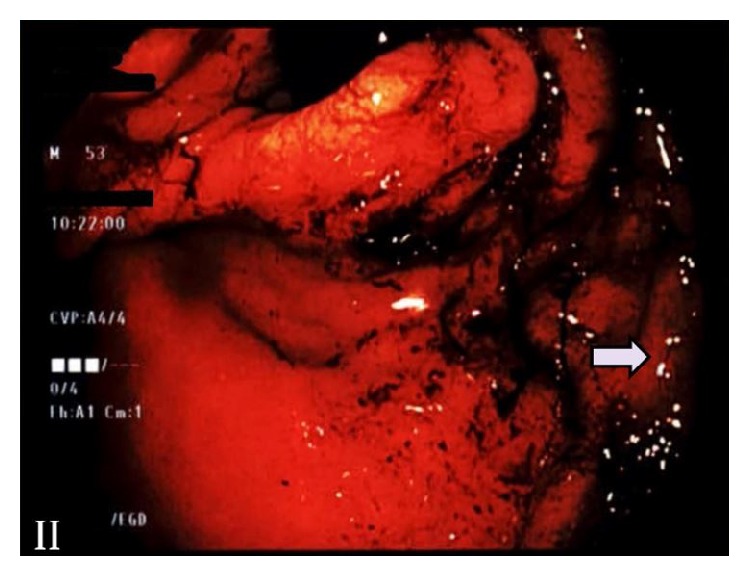
EGD showing retroflex view of GE junction with mass extending from lesser curvature to the cardia with clear demarcation in right lower corner (Arrow), not involving the esophagus.

**Figure 3:**
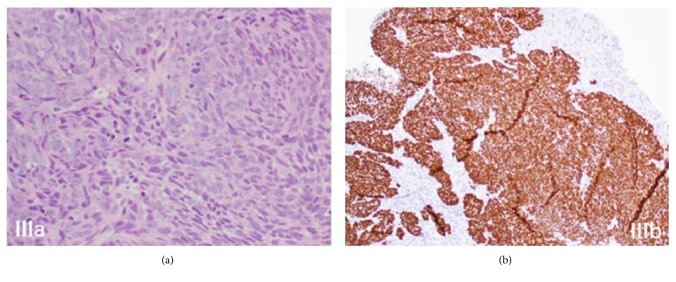
(a) Numerous mitotic figures as well as high grade morphology cells with eosinophilic cytoplasm. (b) P40 Positivity proving squamous differentiation.

Upper endoscopy revealed a large necrotic mass of approximately 6 cm in the lesser curvature with ulceration in the gastric cardia without extension into the esophagus (Figure 2). Biopsies were taken and pathology revealed sections of gastric mucosa with nests of infiltrative neoplastic cells with spindled, vesicular nuclei, irregular nuclear contours, and variably prominent nucleoli. Brisk mitotic activity was noted with focal necrosis, as well as a small focus of keratinization (Figure 3(a)). Immunohistochemical (IHC) stains showed neoplastic cells positive for pancytokeratin including CK7 and CK20, as well as p63 and p40 (Figure 3(b)). Neuroendocrine markers synaptophysin, chromogranin, and CD56 were negative. Pathological diagnosis was made for invasive poorly differentiated squamous cell carcinoma with rare keratinization (Figure 3(a)).

Initially, gastric primary was thought to be less likely initially due to the evidence of keratinization; however, outpatient PET scan showed a 6.5 cm hyper metabolic ulcerative gastric mass and no additional source for a primary tumor. The liver metastasis was appreciated once again, as well as hypermetabolic metastatic adenopathy in the abdomen (Figure 1(b)). A liver biopsy was offered; however, the patient declined.

At this time, he has completed six rounds of systemic chemotherapy including IV Carboplatin and IV Paclitaxel for Stage IV PGSCC with diffuse liver metastasis and gastric lymphadenopathy. He has developed chemotherapy induced thrombocytopenia with platelets of 22,600 K/cmm, and neuropathy treated with gabapentin. Report of the PET scan after 6 months of therapy revealed an enlarging gastric mass, increased adenopathy, and worsening hepatic metastasis.

## 3. Discussion

PGSCC is considerably rare, with incidence reported at 0.2% of all gastric carcinomas [1–6].

Adenocarcinoma accounts for 95% of all gastric malignancies. PGSCC occurs more commonly in middle-aged men [6].

Because many gastric masses, which are initially thought to be PGSCC, ultimately turn out to be of a different cell origin (i.e., gastric adenosquamous cell carcinomas), or from a different location (i.e., extending from the esophagus), criteria developed by Parks et al. exclude any such cases: Originates in the gastric cardia, extends into the esophageal mucosa, or the presence of squamous cell carcinoma from any other site must be ruled out [2, 3]. Prior to the development of Parks et al. criteria, Boswell and Helwig developed histopathologic criteria, where one of the following must be met: (1) Keratinized Cell masses forming keratin pearls, (2) Mosaic cell pattern, (3) Intercellular bridges or (4) Presence of Keratin or Cytokeratin [4].

In respect to these diagnostic criteria, our patient's findings of gastric mass originating in the lesser curvature without extension into the esophagus (Figure 2), PET imaging ruling out another primary site, and histopathologic findings support the diagnosis of PGSCC.

**Figure 4:**
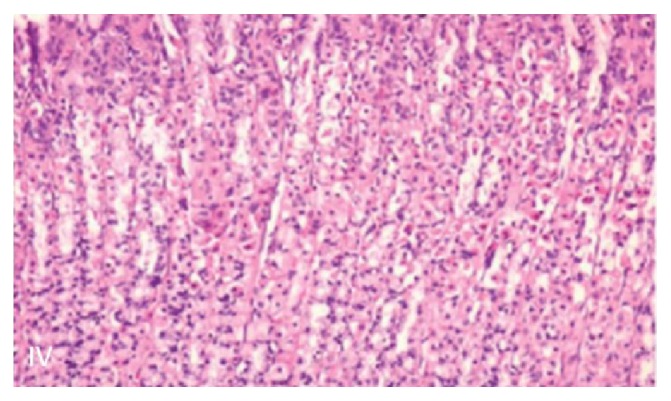
Typical gastric mucosa with normal appearing mucus secreting glands.

P63 tumor marker has been shown to be positive in 81% of squamous cell carcinomas [7] (Figure 3(a)). Moreover, P40 positivity has been shown to improve diagnostic yield for squamous cell carcinoma in poorly differentiated carcinomas, as well as differentiate squamous cell carcinoma from adenocarcinoma [8–10](Figure 3(b)). We provide a sample image of normal appearing gastric mucosa with typical mucus producing glands as a means of comparison to our pathology images of invasive squamous epithelium with high mitotic activity [11] (Figure 4).

The presentation is variable and non-specific. Symptoms include abdominal discomfort, dysphagia, melena, hematochezia, and weight loss. SIADH has not been reported in a PGSCC previously; however, it has been seen in other sites of squamous cell carcinomas [12]. A hyponatremia workup was performed including Urine Osmolality (353 mOsm/kg) and Urine Sodium (<20 mmol/L). These findings along with the patient's clinical picture suggested a multifactorial etiology including hypovolemia, poor solute intake, as well as excess ADH secretion from gastric tumor.

Given its rarity, the etiology is still not well understood. Several theories exist: gastric mucosal stem cells, ectopic squamous epithelium in the gastric mucosa, squamous differentiation of preexisting adenocarcinoma, origin from endothelium of gastric vessels, and squamous metaplasia of glandular epithelium induced by noxious agents [13]. Given our patient's significant history of tobacco and alcohol use, he was at a greater risk of developing squamous cell carcinoma [14]. Along with smoking history, white race and male gender did preclude a higher risk of gastric cancer [15].

When disease is local, radical resection is the therapy of choice. The benefit of adjuvant chemotherapy remains uncertain [16]. Due to the extent of diffuse metastases, chemotherapy was initiated in our patient. The prognosis tends to be very poor across all stages in comparison to gastric adenocarcinoma with a median overall survival of 8 months vs. 19 months [1]. This is most likely due to the commonly involved lymphovascular system, propensity for poorly differentiated tumor grade and late disease state at the time of diagnosis—of note, each of these were factors in our case [17].

While PGSCC is a rare disease, it is vital to keep this in mind when evaluating gastric pathology. With surgical resection being the recommended therapeutic approach, prompt identification with attention to the strict diagnostic criteria is essential.
